# Burgers, brains, and evolution: biological and philosophical roots of fat craving

**DOI:** 10.1038/s41366-026-02029-y

**Published:** 2026-02-04

**Authors:** Mohammed Ayyad

**Affiliations:** Department of Medicine, Rutgers New Jersey Medical School, Newark, NJ USA

**Keywords:** Risk factors, Obesity

## Background

The story of human evolution is often told through tools, fire, and language. Yet food, particularly fat, was equally decisive in shaping the human brain. The same lipid-driven adaptations that once supported cortical expansion and cognitive sophistication enabling the rise of civilization, now drive maladaptive eating in environments of abundance. As global societies confront escalating rates of obesity and metabolic disease, a central question arises: are modern fat cravings a misfiring of once-adaptive instincts, or do they continue to serve a deeper evolutionary role? This Perspective argues that humanity’s relationship with fat represents an outdated evolutionary adaptation that has become maladaptive in conditions of sustained surplus. The craving that is thought to have spurred intelligence may now threaten it. Understanding this paradox may open new avenues for both biomedical research and cultural adaptation.

## Brain Expansion and the Role of Lipids

The human brain is metabolically demanding, consuming a disproportionate share of total energy. Access to lipid-rich foods, particularly long-chain polyunsaturated fatty acids, is believed to have supported cortical expansion and higher-order cognition [1]. Marine and animal fats supplied docosahexaenoic acid and arachidonic acid, both essential for synaptic function and neurodevelopment. Without these nutrients, the remarkable enlargement of the human cortex might not have occurred [2].

Neuroscience confirms that lipids are not only caloric substrates, but also signaling molecules that modulate synaptic plasticity, dopamine release, and endocannabinoid pathways [3, 4]. Therefore, dietary fat functions as both fuel and molecular regulator of cognition. However, it remains unclear whether lipids primarily promote lifelong cognitive health by supporting neuroplasticity and resilience, or whether excess lipid burden more often derails these pathways. Through metabolic dysfunction, inflammatory cytokine signaling, cerebrovascular atherosclerosis, and dysregulated leptin/adiponectin signaling coupled to insulin resistance, excess lipids play a major role in today’s chronic disease processes.

## Evolutionary Preferences and Cravings

While human cravings for fat and sugar were sculpted by scarcity, modern food environments of gustatory super-stimuli increasingly engage dysregulated gut–brain appetite signaling, promoting overnutrition and obesity [5]. In ancestral settings, rare energy-dense foods conferred a survival advantage [6]. Neural reward circuits may have evolved to reinforce the pursuit of such nutrients. The mesolimbic dopamine system linking the ventral tegmental area and nucleus accumbens remains the primary substrate of this drive [7]. Functional neuroimaging demonstrates that high-fat cues robustly activate these regions, particularly in individuals with obesity, reflecting a hyper-reactive reward system [8]. Endocannabinoids and endogenous opioids further amplify hedonic responses, while gut–brain communication via bidirectional leptin, ghrelin, and vagal feedback loops modulates appetite. This complex neurobiological architecture suggests that obesity cannot be reduced to a ‘calories in, calories out,’ framework, because craving is embedded in hardwired gut–brain neurochemistry that resists sole conscious regulation.

## Civilization and the Cognitive Demands of Society

The rise of civilization introduced new cognitive pressures from abstract reasoning to symbolic communication. However, evolutionary change did not keep pace. The human brain has not significantly expanded in the past ten millennia [9]. Instead, culture, language, and environment provided external scaffolding for cognition. This may suggest that modern fat cravings no longer reflect an adaptive nutritional demand for cognitive function but instead represent the persistence of ancient biological drives that have become maladaptive within environments characterized by caloric abundance and highly processed foods.

Mechanistic studies, however, reveal that lipids still influence cognition. Triglycerides and their metabolites can alter reward pathways, impair cognitive flexibility, and reinforce compulsive eating. Genetic variation in fat-sensing receptors such as *CD36*, and in reward-related genes including *FTO* and *OPRM1*, produces population-level differences in fat preference [10]. Epigenetic modifications shaped by prenatal and early-life nutrition further calibrate these pathways and embed vulnerability or resilience within the architecture of appetite and reward.

## The Evolutionary Mismatch of Fast Food

Ultra-processed, lipid-dense foods engineered for hyperpalatability epitomize the collision between ancient instinct and the modern nutritional environment. The craving is authentic, but its modern outlet is pathological. Unlike ancestral lipid sources, contemporary diets are rich in saturated and trans fats that impair cognition, promote inflammation, and accelerate metabolic dysfunction. The paradox is striking in that the same biological drive that once enhanced survival and intelligence may now undermine them.

Comparative biology may already be illustrating this mismatch. Humans and non-human primates share conserved fat-preference pathways, however, humans exhibit especially dense neuropeptide Y innervation within the nucleus accumbens likely creating a predisposition to hedonic overeating [11]. What once supported survival now interacts with engineered food environments to amplify compulsive, reward-driven eating patterns that share features with addiction-like behavior.

## Philosophical Considerations: Are We Still Evolving?

This evolutionary paradox reframes modern nutrition as an ongoing experiment in adaptation shaped by rapid environmental change rather than slow biological evolution. Several priorities emerge for future inquiry. First, in neuro-nutrition, to what extent do contemporary dietary lipid profiles influence neuroplasticity, stress-related neural responses, and trajectories of cognitive aging across the lifespan? Second, in evolutionary biology, how do genetic variation and cultural inheritance interact to shape vulnerability or resilience to hedonic overeating, given that biological selection is unlikely to keep pace with modern food environments characterized by high variability and frequent alterations? Third, in cultural adaptation, which modifications of food environments meaningfully attenuate reward-driven overconsumption at scale, and under what conditions might emerging precision nutrition approaches, including nutrigenomics and gut–brain theraputics, provide additive benefit?

Integrative strategies linking longitudinal human cohorts with multi-omics profiling of microbiota, metabolism, and gut–brain neurochemistry may clarify the biological substrates of fat craving and identify modifiable targets for prevention and treatment. Such approaches are particularly needed to determine how sustained dietary lipid exposure over the life course recalibrates reward circuitry, metabolic flexibility, and long-term resilience to obesogenic environments.

## Conclusions

Fat is both a nutrient and a narrative, a biological driver that once sustained life and now, under conditions of persistent surplus, increasingly contributes to disease. The central issue does not reside in the presence of craving itself, but in the mismatch between evolutionarily conserved reward mechanisms and the modern nutritional environment in which they operate. What evolved as a mechanism for survival now operates in settings defined by engineered fats and hyperpalatable foods, where ancient instincts collide with abundance and contribute to obesity, chronic inflammation, and cognitive vulnerability. This mismatch between biology and environment represents one of the most consequential public health challenges of the modern era.

This Perspective identifies fat craving as a residual evolutionary program that shapes susceptibility to hedonic overeating without determining behavior, moving beyond interpretations grounded solely in willpower or personal failure. Recognizing fat craving as a neurobiological and evolutionary construct may open new avenues for intervention while preserving substantial room for individual agency, cultural adaptation, and policy-level change. Future research should delineate lipid-driven signaling along the gut–brain axis, as the success of GLP-1-based therapies already demonstrates the power of modifying hardwired appetite neurochemistry by suppressing excess food cravings to treat obesity. Genetic and epigenetic profiling may further clarify susceptibility patterns that predispose to maladaptive craving and enable more precise, personalized nutritional strategies. At the population level, efforts should shift from reactive management and calorie-centered models to intentional design, reshaping food environments to be informed by satiety biology, and embedding neurobehavioral science into dietary guidance to nudge healthier food choices.
